# Research Trends and Evolution of Astrocytes in Depression and Antidepressant Treatment: A Bibliometric Analysis

**DOI:** 10.2174/011570159X353752250227113751

**Published:** 2025-03-18

**Authors:** Shu-Man Pan, Zhe Li, Jing-Qi Zhou, Xiang Shang, Tian-Jia Gu, Xiao-Ming Sun, Zhen-Hua Zhu

**Affiliations:** 1 Affiliated Guangji Hospital of Soochow University, Suzhou, Jiangsu Province, 215137, P.R. China

**Keywords:** Astrocyte, depression, antidepressant treatment, bibliometric analysis, VOSviewer, neuroinflammation

## Abstract

**Background:**

Astrocytes have emerged as key players in the pathogenesis of depression and antidepressant treatment. However, comprehensive reviews in this field were absent. The bibliometric analysis can effectively illustrate research trends and hotspots of a specific domain through analysis of publications.

**Objective:**

We conducted a bibliometric analysis to overview the current hotspots and research trends of astrocytes in depression and antidepressant treatment.

**Methods:**

We collected publications’ data from the science citation index expanded (SCI-E) of the Web of Science (WOS) database, and bibliometric analysis was applied through CiteSpace and VOSviewer software. Results were mapped *via* GraphPad Prism, Adobe Photoshop, and R software.

**Results:**

After analysis of 2896 publications, we analyzed the content of publications, most influential publications, productive journals, most cited journals, core authors, productive countries/regions, and institutions in this field. The cooperation of main countries and organizations was mapped. Most importantly, after a thorough analysis of keywords, we found neuroinflammation was a hot topic in this research field.

**Conclusion:**

The results of the bibliometric study prove neuroinflammation is a hot topic in this research field. Nowadays, many studies have investigated the role of astrocytes in depression and antidepressant treatment from the perspective of neuroinflammation. It is essential to pay more attention to elucidating the mechanisms of astrocyte-mediated neuroinflammation to identify potential targets for antidepressant development.

## 1 INTRODUCTION

Depression, mainly referred to as Major Depression Disorder (MDD), also named unipolar depression, is a common mental disorder characterized as anhedonia. It is a major cause of disability worldwide and imposes a significant economic burden [1]. Nowadays, more than 700,000 people commit suicide due to depression per year [2]. World Health Organization (WHO) estimated that depression would be the leading cause of disease burden by 2030 [2, 3]. However, despite extensive research, the pathogenesis of depression remains unclear [4]. The treatment of depression is still challenging due to its unclear pathogenesis [5]. Therefore, it is urgent to illustrate the pathogenesis of depression and identify new antidepressant targets.

Astrocytes are the most abundant glial cells in the brain and play an important role in maintaining Central Nervous System (CNS) homeostasis, such as sustainment of Blood-Brain-Barrier (BBB) [6], regulation of glucose metabolism [7], secretion of gliotransmitters [8]. Besides, astrocytes are the main component of the glymphatic system [9] and play an important role in the process of neuroinflammation [10]. Accumulating evidence demonstrates that astrocytes are involved in the pathogenesis of depression and other mood disorders [9]. Glial Fibrillary Acidic Protein (GFAP) is a marker of astrocytes. A decrease of GFAP-positive cells was observed in the prefrontal cortex [11, 12], cingulate cortex [13], thalamus [14], and amygdala [15, 16] of depressed suicides. Postmortem studies also found hypertrophy of astrocytes in BA24 white matter in patients with depression [17]. Similar numbers and morphological changes of astrocytes also appeared in depressive animal models [18, 19]. Moreover, the ablation of prefrontal cortex astrocytes can induce depression-like behaviors in mice [20]. Importantly, antidepressants have been shown to modulate astrocytic function, which might underlie their therapeutic effects [21, 22]. The above-mentioned evidence demonstrates astrocytes contribute to the pathogenesis of depression and the mechanism of antidepressant treatment. Therefore, it is significant to comprehensively analyze the astrocytes-related research literature in depression and antidepressant treatment field.

Bibliometric analysis is a powerful tool for quantitatively evaluating the research trends of a specific field [23]. For example, Bai *et al.* proved that antioxidant activity was the hotspot of natural polysaccharides through bibliometric analysis [24]. Thus, in this study, we conducted a bibliometric analysis based on the 2896 publications from the Web of Science database. We mapped the research trends in this research field and confirmed neuroinflammation was a hot topic. Then, we reviewed the dual effect of astrocytes in CNS inflammation modulation, highlighting the role of astrocyte-mediate inflammation in depression development and antidepressant treatment. Moreover, we focused on the mechanism by which astrocytes govern neuroinflammation in depression. The main structure of this study is shown in Figs. (**1a** and **1b**). These results help to provide a comprehensive overview of the research trends and identify potential research directions for future studies in this research field.

## 2 MATERIALS AND METHODS

### 2.1 Data source

Publications up to September 30, 2024, were searched from the science citation index expanded (SCI-E) of Web of Science (WOS) database using the following strategy: TS = (Astrocyte OR Astrocytes OR Astroglia OR Astroglias OR “Astroglial Cells” OR “Astroglia Cell” OR “Cell, Astroglial” OR “ Astroglia Cells” OR “Astroglia Cell” OR “Cell, Astroglia”) AND TS = (Depression OR “Depressive Symptoms” OR “Depressive Symptom” OR “Symptom, Depressive” OR “Emotional Depression” OR “Depression, Emotional” OR “Antidepressant Drug” OR “Drug, Antidepressant” OR Antidepressants OR Antidepressant OR “Antidepressant Drugs” OR “Antidepressant Medication” OR “Medication, Antidepressant” OR “Antidepressive Agent” OR “Agent, Antidepressive” OR “Thymoanaleptics” OR “Thymoanaleptic” OR “Thymoleptics” OR “Thymoleptic”).” The search was limited to documents written in English and published in peer-reviewed journals (PRISMA flowchart). The published time was set from 1900-01-01 to 2024-09-30. To avoid bias caused by the frequent updates of the database, all data retrieval and collection were completed in one day on October 1, 2024. The earliest literature we retrieved in the research field of astrocytes and depression was published in 1967. A total of 2379 articles and 517 reviews were included for analysis. It is worth mentioning that some journals classify meta-analysis as an article, while others categorize it as a review (Supplementary data).

### 2.2 Bibliometric Analysis

The data of publication numbers and journals of this field were directly acquired from the Web of Science. The content of the publication was evaluated and classified by reading the abstract. The core authors, top 15 cited documents, top 10 productive journals, most productive countries/organizations, and cooperation of countries/organizations were analyzed through VOSviewer 1.6.19. The occurrence and clustering of author-defined keywords were identified through VOSviewer [25]. The top 10 keywords with the strongest citation bursts and timelines for keywords were analyzed by CiteSpace 6.1 R3 [26]. For keywords analysis, synonym terms were manually merged, for example, “astrocyte” and “astrocytes,” “CUMS” and “chronic unpredictable mild stress.” Figures were drawn by Prism 7 (GraphPad Software), Adobe Photoshop, and R 4.2.3 software, including package ggplot2 3.5.1 and circlize 0.4.16.

## 3 RESULTS

As shown in Fig. (**2**) and Table **S1**, publications of astrocytes and depression increased steadily, which indicated that more researchers began to concentrate on this field.

### 3.1 Publication

#### 3.1.1 Publication Content

**Fig. (3):**
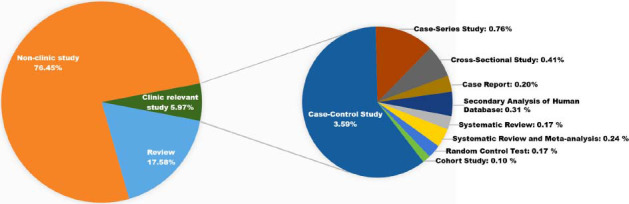
Content of publication in the research field of astrocytes and depression. In this figure, “non-clinic study” refers to *in vivo*, *in vitro*, *in silico* experiments, and other research. “Review” refers to reviews that exclude clinic-relevant systematic reviews.

Firstly, we analyzed the content of publications through reading abstracts. About 76.45% of publications are non-clinic studies, including *in vivo, in vitro, ex vivo, in silico* researches (Table **1** and Fig. **3**). Then, we conducted a detailed classification of clinic-relevant studies. As shown in Table **1** and Fig. (**3**), clinic publications in this field are mainly case-control clinic studies.

#### 3.1.2 High-Cited Publications

The number of citations could reflect the impact of publications. High-cited papers always represent cutting-edge research results in this field and guide future research directions. Here, we summarized publications with high citations in this field and sorted them by citation frequency (Table **2**). Among the 15 core publications, there are 8 research articles and 7 reviews.

The most influential research was conducted by Banasr *et al*., who proved that pharmacologic ablation of the Prefrontal Cortex (PFC) glia in adult rats could induce depression-like behaviors [20]. Similarly, Cao *et al.* revealed that insufficient ATP levels in the brain was tightly linked to depression and that stimulating endogenous ATP release from astrocytes could relieve depression-like behaviors of animal models [27]. This work is very influential, and many people have followed in their footsteps to conduct research. Substantial evidence from animal studies proved that a decrease in ATP release from astrocytes might participate in the development of depression [28, 29], and clinical antidepressants also increase the level of astrocyte-released ATP *in vivo* [30]. Cui *et al*. also conducted meaningful and impactful research, demonstrating that astrocytic Kir4.1 in the lateral habenula is essential for driving depression-like behaviors [31]. In this research field, high-cited reviewers and articles mainly focus on the changes in astrocytes under depressive states [16, 32-34] and the way that astrocytes are involved in the pathogenesis of depression [34-41].

### 3.2 Journal

According to the analysis results of VOSviewer, the top 10 productive journals in this research field are summarized in Table **3**. Brain Research was the most productive journal (66 publications), and the Journal of Neuroscience was the most cited Journal (9018 citations) in the study of astrocytes and depression.

### 3.3 Author

Author analysis in Table **4** shows the 10 core authors in the field of astrocytes and depression. Li BaoMan from the China Medical University and Verkhratsky Alexei from the University of Manchester were the most prolific authors, followed by Peng Liang from the China Medical University. Araque Alfonso from the University of Minnesota was the most influential author, with 2946 citations in this research field. Notably, researchers in this field have kept in tight cooperation, the cooperation map of authors was provided in supplementary materials (Fig. **S1**).

### 3.4 Countries/Regions and Organizations

**Fig. (4):**
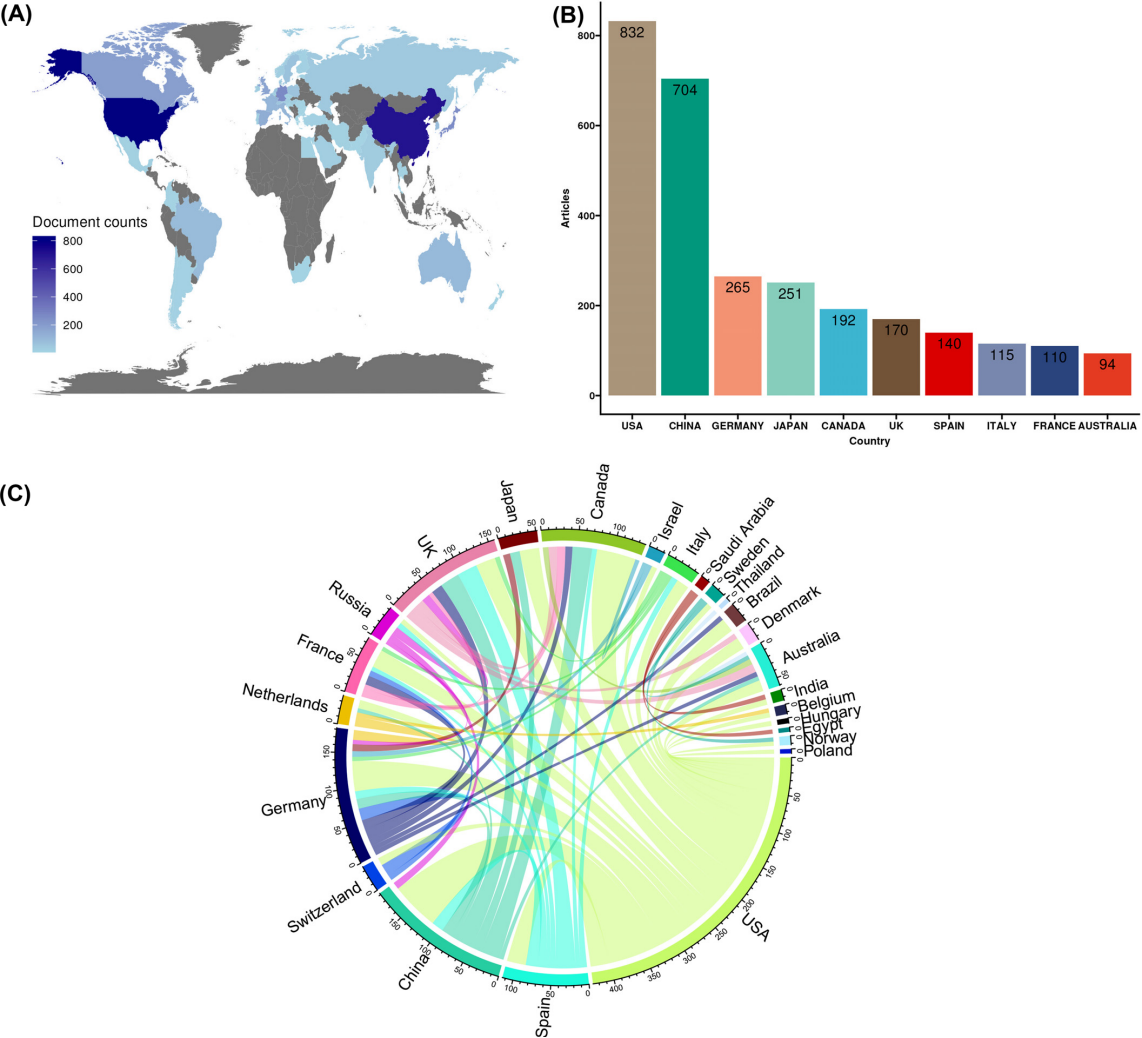
Overall analysis the countries’ information in the research field of astrocytes and depression. (**A**) Spatial distribution of publications in astrocyte and depression research field. (**B**) Number of publications by countries/regions. (**C**) Visualize the cooperation relationships of countries/regions. In our study, countries/regions with not less than 5 publications were selected for analysis.

The spatial distribution of publications and detailed publication number of different countries/regions is recorded in (Table **5** and Fig. **4**). The USA was not only the country with the highest number of publications (832, 28.73%) but also the country with the most citations (Table **5**; Figs. **4A**, **B**). At the same time, the USA also has close cooperation with other countries/regions in this research field. The cooperation maps of countries are shown in Fig. (**4C**) and supplementary materials (Fig. **S2**). The role of astrocytes in depression and antidepressant treatment is a globally concentrated question. China, Germany and Japan also published a lot of papers in this research field (Table **5**; Figs. **4A**, **B**). Many countries/regions tightly collaborated to illustrate the way that astrocytes are involved in depression pathogenesis and antidepressant treatment (Fig. **4C** and Fig. **S2**).

We also analyzed the publication numbers and cited times of organizations in this research field. The top 5 most prolific organizations were China Medical University, McGill University, University of Toronto, University of Manchester, and Consejo Superior de Investigaciones Cientificas. Academic organizations that focused on this field are mainly distributed in China, the United States, Canada, Japan and Europe (Table **6**), which was consistent with the analysis results of countries. Consejo Superior de Investigaciones Cientificas was the most cited organization in this research field (Table **6**). And University of Manchester has the most active cooperation with other academic organizations. The cooperation of organizations was visualized in supplementary materials (Fig. **S3**).

### 3.5 Keywords Analysis

**Fig. (5):**
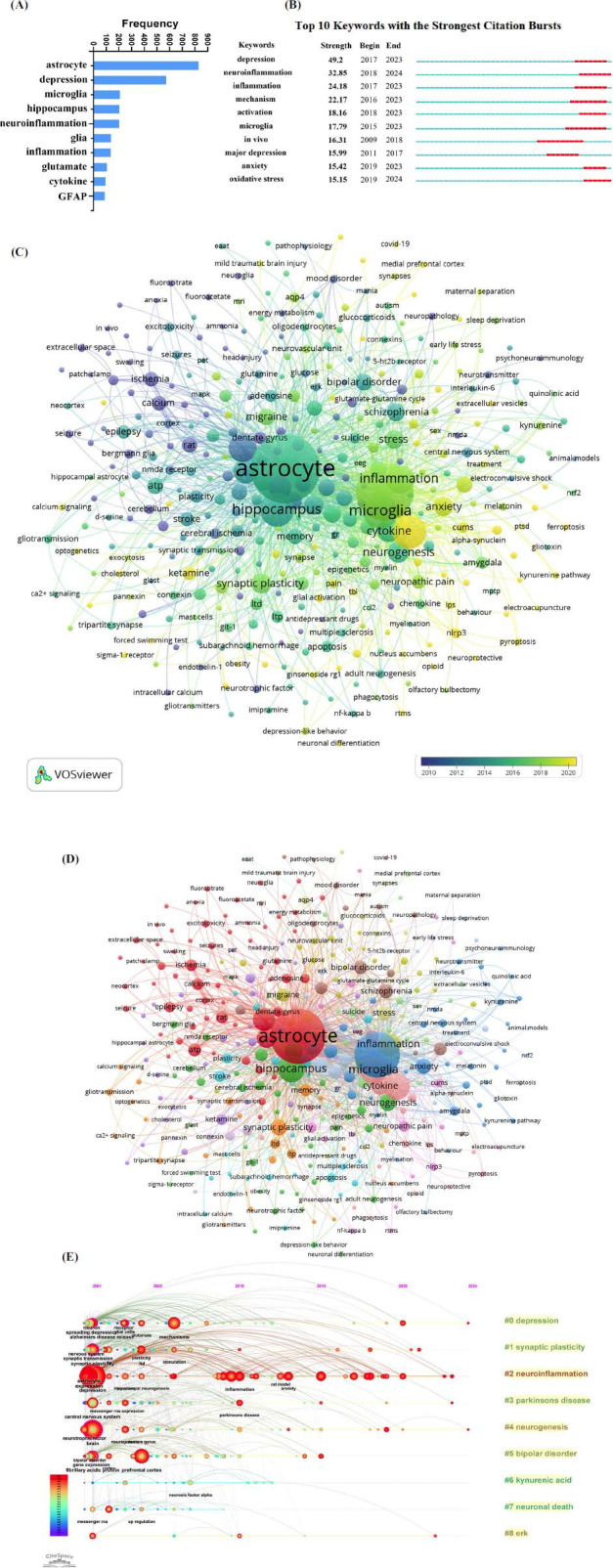
Overall analysis of author-defined keywords on astrocytes and depression research field. (**A**) The frequency of author-defined keywords in this field. (**B**) Top 10 keywords with the strongest citation bursts in this field. The blue line represents the timeline, and the red portion on the blue timeline indicates the time of the keyword outbreaks. This analysis was processed through Citespace. (**C**) Author keywords were displayed as different colors according to the time of their occurrences. As indicated by the color bar, keywords that appeared earlier are represented in blue, while keywords that appeared later are represented in yellow. (**D**) The occurrence and cluster network of the author's keywords in the field of astrocytes and depression (391 keywords, frequency >5). (**E**) The keywords timeline view in the field of astrocytes and depression. The position of the nodes indicates their appeared time, and the size of the nodes is positively correlated with the frequency of keywords. The lines between different nodes represent the co-occurrence relationships between the keywords. As the color bar shows, keywords that appeared earlier are represented in blue, while keywords that appeared later are represented in red.

According to the bibliometrics theory, keyword analysis helps to illustrate the hotspot and offer directions for future study of this research field [42]. In this study, we analyzed the frequency of author-defined keywords. Of 5349 keywords, the top 10 frequent keywords are astrocyte, depression, microglia, hippocampus, neuroinflammation, glia, inflammation, glutamate, cytokine, and GFAP (Fig. **5A**). Most of them are associated with CNS immune status and related to the pathogenesis of depression.

Citation bursts mean that keywords are highly cited in a given period, which always represents breakthroughs or emerging topics in a specific field. It is worth mentioning that neuroinflammation, inflammation, activation, microglia, and oxidative stress have had strong citation bursts in recent years (Fig. **5B**). The Average Appearance Year (AAY) is defined as the average time derived from the integration of the first and last occurrences of a specific keyword, which could indicate the relative novelty of the keyword. In this study, we analyzed the AAY of keywords and overlaid their occurrences on a timescale through VOSviewer (Fig. **5C**). Through analysis of AAY of high-frequency keywords, we found GFAP (keyword AAY 2010.5), glia (keyword AAY 2011.4), and glutamate (keyword AAY 2013.7) were the primary hotspots. Inflammation (keyword AAY 2017.8) and neuroinflammation (keyword AAY 2020.5) were the recent focus of this research field. Then, the co-occurrence of the keywords network was mapped through VOSviewer to visualize inflammation-related factors in the field of astrocytes and depression (Fig. **5D**). Furthermore, we performed a temporal analysis to delineate the chronology of keywords related to astrocytes and depression. As illustrated in Fig. **5E**, clusters #5 (bipolar disorder), #6 (kynurenic acid), #7 (neuronal death), and #8 (ERK) are no longer regarded as research hotspots in this field. In contrast, cluster #2 (neuroinflammation) contains more highly emergent keywords and has become a very popular research direction in recent years. Both keywords frequency, citation burst analysis, AAY calculation, and keywords timeline view prove that neuroinflammation has been the research frontier of astrocytes and depression in recent years.

## 4 DISCUSSION

### 4.1 Research Trends in Astrocyte and Depression

There is no doubt that astrocytes play a crucial role in the pathogenesis of depression and the efficacy of antidepressant treatments. To gain deeper insights into this field, we conducted a bibliometric analysis based on 2896 publications from the WOS database. In this field, basic research significantly outnumbers clinical research. Furthermore, most clinical studies are low-level case-control studies. Moving forward, there is a pressing need for more high-quality Randomized Controlled Trials (RCTs) and cohort studies to advance this area.

Our analysis of highly influential literature indicated that, in recent years, most high-cited papers were primarily focused on neuroinflammation, aligning with the findings of our keyword analysis. Additionally, the analysis of core authors, top productive countries/regions, and institutions revealed that the United States and China were the leading countries in terms of publication numbers and citation frequency, serving as the primary hubs for key institutions and authors.

### 4.2 Hotspot in Astrocyte and Depression: Neuroinflammation

The concept that depression is intertwined with chronic low-grade inflammation has gained substantial attention in recent years. It is well-recognized that inflammation is a risk factor for MDD [43]. Cumulative studies have demonstrated peripheral proinflammatory markers, such as CRP, IL-6, IL-1β, and TNF-α, were significantly increased in individuals suffering from depression [44, 45]. A comprehensive meta-analysis revealed that levels of IL-6 and TNF-α were significantly elevated in the Cerebrospinal Fluid (CSF) of MDD patients. Additionally, increased TNF-α level was also observed in the post-mortem brains of MDD patients [35]. Furthermore, sirukumab (a tumor necrosis factor antagonist) produced better antidepressant activity than placebo in depressive participants with CRP concentration > 5 mg/L [46].

For depression-related neuroinflammation, previous studies mainly focused on microglia [33, 47], the resident immune cells of CNS. However, astrocytes are also involved in the maintenance of CNS immune homeostasis. More and more studies have found astrocytes contribute to CNS inflammation in depression. In our study, the results of keyword co-occurrence network, keyword burstness, and keyword timeline view (Fig. **5**) proved neuroinflammation has emerged as a hot topic in the research field of astrocytes and depression in recent years. Thus, based on the results of bibliometric analysis, we conducted a thorough review to illustrate how astrocyte-mediated neuroinflammation contributes to depression and the effectiveness of antidepressant treatment.

### 4.3 In-depth Review: The Role of Astrocytes in the Pathogenesis of Depression and Antidepressant Treatment through Neuroinflammation

#### 4.3.1 The Dual Role of Astrocytes in Neuroinflammation

Astrocytes exhibit a high degree of heterogeneity and play diverse roles in the regulation of neuroinflammation. On one hand, they play a crucial role in dampening inflammation and providing neuroprotective effects [48]. Conversely, astrocytes can also release pro-inflammatory cytokines, trigger the activation of microglia, recruit peripheral immune cells, and intensify the inflammatory response within the CNS [49].

The plasticity of astrocytes allows them to transfer to different phenotypes depending on the surrounding conditions. Presently, it is widely acknowledged that reactive astrocytes can be categorized into two main phenotypes: pro-inflammatory and anti-inflammatory. Pro-inflammatory astrocytes, also known as A1 astrocytes, are typically stimulated by Pathogen-Associated Molecular Patterns (PAMPs) such as Lipopolysaccharide (LPS) [50, 51] and certain pro-inflammatory cytokines [52-54]. Anti-inflammatory A2 astrocytes are induced by factors such as TGF-β [55, 56] or events like ischemic damage [57]. Studies utilizing gene transcriptome analysis confirmed that A1 astrocytes were C3-specific positive [52, 58-60], while S100A10 is uniquely expressed in A2 astrocytes [58, 60, 61]. Zou *et al.* have identified significant morphological differences between these two astrocyte subtypes. C3+ A1 astrocytes exhibit elongated dendrites, while S100A10+ A2 astrocytes show a reduced number of dendritic processes, both *in vivo* and *in vitro* [62]. We summarized the key characteristics of the classic astrocyte phenotypes in Table **7** [63-81].

#### 4.3.2 Astrocytes Involved in Depression-related Neuroinflammation

The current studies indicate that astrocytes in depression predominantly exhibit pro-inflammatory A1 characteristics. Xie *et al.* examined the levels of inflammatory cytokines in astrocyte-derived extracellular vesicles (ADEs) obtained from 70 patients diagnosed with MDD and healthy controls. Their findings demonstrated a significant increase in inflammatory markers, including IFN-γ, IL-1β, IL-6, TNF-α, and IL-17A, in the ADEs from MDD patients [82]. Moreover, FAM19A5, a novel marker associated with reactive astrogliosis and neuroinflammation [83], was found to be significantly elevated in depression patients [84], indicating an inflammatory phenotype of astrocytes in depressive conditions.

The activation of astrocytes was also observed in animal models of depression. Uncoupling protein 2 (UCP2), located in the inner membrane of mitochondria, plays a crucial role in restricting the production of Reactive Oxygen Species (ROS). Knockout UCP2 could lead to the activation of the nod-like receptor protein 3 (NLRP3) inflammasome in hippocampal astrocytes and induce depression-like behaviors in mice [85]. Overexpression of IL-33 in amygdala astrocytes has been demonstrated to directly induce anxiety-like behaviors in mice [86]. The above-mentioned evidence substantiates the pivotal role that inflamed astrocytes play in the pathogenesis of depression.

Targeting astrocytes could relieve neuroinflammation and produce an antidepressant effect. Specific overexpression of the sigma-1 receptor (Sigma-1R) in hippocampal astrocytes has been shown to ameliorate depressive-like behaviors in mice injected with LPS [87]. Furthermore, administration of calpain inhibitors has been found to alleviate the activation of hippocampal astrocytes and prevent depression-like behaviors in animal models induced by Chronic Unpredictable Mild Stress (CUMS) and LPS [88].

Currently, the most commonly used antidepressants in clinical practice are Selective Serotonin Reuptake Inhibitors (SSRIs) and Serotonin-Norepinephrine Reuptake Inhibitors (SNRIs). SSRIs, including fluoxetine, paroxetine, sertraline, fluvoxamine, citalopram, and escitalopram, are widely regarded as the first-line treatment for depression. These medications are designed to inhibit the reuptake of serotonin (also known as 5-hydroxytryptamine, 5-HT), thereby increasing serotonin levels in the brain and alleviating depression-like behaviors. Notably, research has shown that SSRIs not only inhibit serotonin reuptake but also play a significant role in modulating astrocyte activation. For instance, fluoxetine has been demonstrated to inhibit astrocyte activation both *in vivo* and *in vitro* [59]. Paroxetine has been shown to ameliorate reactive microglia-induced inflammatory responses in astrocytes, partially by inhibiting the NF-κB (nuclear factor kappa-light-chain-enhancer of activated B cells) pathway [51]. A cytokine mixture of C1q, TNFα, and IL-1α can stimulate the release of IL-6 and IL-1β in primary astrocyte cultures. Pre-treatment with SSRIs significantly suppresses the production of these pro-inflammatory cytokines [89]. Venlafaxine, the representative drug of SNRIs, significantly reduced the production of IL-1β and IL-6 in cytokine mixture-stimulated astrocytes [89]. Other antidepressants, such as trazodone, have been found to activate the AKT pathway and reduce the release of pro-inflammatory cytokines in LPS and TNF-α-stimulated astrocytes [90].

In addition to clinically used antidepressants, many natural products have been shown to alleviate astrocyte-mediated inflammation and improve depressive-like behaviors in animal models. Scutellarin improved depression-like behaviors of mice injected with LPS as well as inhibited Toll-Like Receptor 4 (TLR4)/NF-κB pathway in LPS-treated astrocytes [91]. *Morinda officinalis* oligosaccharides suppressed the secretion of IL-6, IL-1β, and TNFα in LPD-stimulated astrocytes and exerted antidepressant activity in hypertension rats [92]. Yomogin, a compound isolated from *Artemisia iwayomogi*, reduced the number of activated astrocytes and alleviated neuroinflammation in LPS-injected mice [93]. Quercetin-enriched diet attenuated depressive-like behaviors of Chronic Social Defeat Stress (CSDS) model by inhibiting astrocyte reactivation [94]. Xanthohumol, a bioactive prenylated flavonoid, relieved LPS-induced depressive-like symptoms as well as suppressed activation of astrocytes in the hippocampus of mice [95]. Folic acid, a medication used for pregnancy, promoted astrocytic IL-10 expression by inhibiting EZH2-mediated H3K27me3 and improved depression-like behaviors in adult mice [96].

These findings highlight the critical role of astrocyte-mediated neuroinflammation in the pathogenesis of depression. Furthermore, they suggested targeting or alleviating the over-activation of astrocytes might be an effective strategy for developing new antidepressant treatments.

#### 4.3.3 The Mechanisms Underlying Astrocyte-mediated Neuroinflammation

To effectively target astrocyte-related inflammation, it is crucial to elucidate the mechanism underlying astrocyte-mediated neuroinflammation. Therefore, we analyzed key factors and intracellular signaling pathways that regulate this process. Our analysis revealed various molecules associated with astrocytic inflammation (Figs. **3C**, **D**), including NLRP3 (23 occurrences), NF-κB (16 occurrences), Nuclear Respiratory Factor 2 (Nrf2, 7 occurrences), indoleamine 2,3-dioxygenase (IDO, 6 occurrences), Mitogen-Activated Protein Kinase (MAPK, 5 occurrences), TLR4 (5 occurrences), Signal Transducer And Activator Of Transcription (STAT, 4 occurrences) and several cytokines (Figs. **3C**, **D**). Notably, the activation of NF-κB has been shown to upregulate the expression of both NLRP3 and proinflammatory cytokines [97]. These results prove that NF-κB is a central regulator in the inflammatory response mediated by astrocytes.

Key components of the NF-κB signaling pathway include NF-κB dimers, IκB (inhibitor of κB), and IKK (the IκB kinase) complex. Under physiological conditions, most NF-κB dimers, typically composed of the p50 and p65 subunits, are trapped in the cytoplasm by IκB proteins. In the canonical NF-κB activation pathway, various stimuli like cytokines (*e.g*., TNF-α, IL-1β), microbial products, and stress signals activate specific receptors, which would lead to the activation of the IKK complex. Once activated, the IKK complex phosphorylates IκB proteins, resulting in the degradation of IκB. Then, NF-κB dimers are released and translocate into the nucleus, initiating the transcription of pro-inflammatory genes [98-102].

Current researches show that the activation of the NF-κB pathway in astrocytes is involved in the pathogenesis of depression. Clinic study found patients with loss-of-function multiple endocrine neoplasia type 1 (Men1; protein: menin) mutations had higher levels of anxiety, depression, and fatigue compared with the general population [103]. Leng *et al.* proved that menin deficiency promoted NF-κB activation and IL-1β activation of astrocytes and specifically knockout astrocytic Men1-induced depressive-like behaviors of mice [41]. Zhang *et al.* reported kynurenine treatment stimulated NF-κB to translocate into the nucleus and bind to the promoter of NLRP2 to stimulate its transcription in astrocytes. Intraperitoneal injection of kynurenine activated astrocytic NLRP2 inflammasome and induced depression-like behaviors in mice [104].

Many studies have proved that targeting the astrocytic NF-κB pathway could improve depression-like behaviors in various animal models. Blockage of two-pore domain potassium channel TWIK-1 could inhibit astrocyte overactivation by suppressing NF-κB signaling pathway and alleviate depression-like behaviors of CUMS rats [105]. Crystalline silica exposure induced astrocyte activation and depression-like behaviors in mice. Nicotine could reduce crystalline silica exposure-induced astrocyte activation by weakening NF-κB signaling and attenuating the abnormal behaviors of mice [106]. Ginsenoside Rb1 modulated the NF-κB pathway to inhibit astrocytic pyroptosis in CUMS rats [107]. Gypenoside-14 downregulated the astrocytic NF-κB signaling pathway *in vitro* and inhibited astrocyte overactivation in LPS-stimulated depressive mice [108]. These findings prove that regulating astrocytic NF-κB signaling pathway is very important for the treatment of depression.

According to previous studies, the modulation of the NF-κB signaling pathway is mainly based on the regulation of IKKs or the ubiquitination process [102, 109]. Ye *et al.* found class IIa histone deacetylases 7 (HDAC7) was selectively increased in LPS-stimulated astrocytes *in vivo* and *in vitro*. HDAC7 could bind to IKK to promote its activation and lead to the nuclear translocation of NF-κB [110]. Aryl hydrocarbon receptor (AHR) could limit NF-kB signaling through the suppressor of cytokine signaling 2 (SOCS2) [111, 112] and directly dimerize with the NF-kB subunits [113]. Specific inactivation of astrocytic AHR increased the expression of pro-inflammatory molecules and worsened Experimental Autoimmune Encephalomyelitis (EAE) symptoms of mice [111]. Nowadays, NF-κB signaling pathway is the most extensively studied pathway in the immune regulation process of astrocytes. However, how to modulate the astrocytic NF-κB signaling pathway to relieve depression-related neuroinflammation still needs to be investigated.

Apart from NF-κB, Janus kinases (JAK)/STAT and other signaling pathways also play important roles in the regulation of astrocyte-related neuroinflammation. In astrocytic cytoplasm, β-arrestin2 could combine with STAT3 and inhibit the activation of the JAK/STAT3 pathway to relieve neuroinflammation of depression animal models [114]. Recent studies verified Sigma-1R receptor was involved in the pathogenesis of depression [115]. Specific stimulation of astrocytic Sigma-1R significantly improved depression-like behaviors of LPS-treated mice [87]. It is worth mentioning that there is a crosstalk between different signaling pathways in the regulation of astrocytic immune process [110, 116, 117].

#### 4.3.4 Future Directions in this Research Field

The Average Appearance Year (AAY) can indicate the relative novelty of specific keywords. The keywords with both relatively recent AAYs and high-frequency have the potential to be future directions. In our analysis, the following keywords may represent emerging trends in this field: glymphatic system (5 occurrences, AAY 2023), ferroptosis (7 occurrences, AAY 2022.9), pyroptosis (10 occurrences, AAY 2022.2), extracellular vesicles (7 occurrences, AAY 2022.1), gut-brain axis (7 occurrences, AAY 2022), and postpartum depression (5 occurrences, AAY 2022).

However, ultimately, the most appealing research direction is to achieve clinical benefit by reprogramming astrocytic activity in depression. Studies have shown that TGF-β or FGF can promote the conversion of A1 astrocytes to the A2 phenotype *in vitro* [52]. Certain compounds, such as MCC950, matrine, and H_2_S, have been proven to inhibit A1 astrocytes or stimulate A2 astrocytes [118]. However, to date, no effective strategy has been directly identified to specifically alter the activation state of astrocytes within living organisms. Moreover, the Brain microenvironment is too complex. Astrocytes continuously secrete signaling molecules, interact with neighboring cells, and exchange materials with their surroundings. How stable are astrocytes activation states in depression is confusable. Understanding the key pathways that shift astrocytes from different phenotypes remains a compelling and important question in this research field. Greater efforts are needed to clearly illustrate the phenotype and immune status of astrocytes at different stages of depression.

### 4.4 Strength and Limitations

To the best of our knowledge, this is the first bibliometric analysis conducted in the field of astrocytes and depression. We mapped the research trends and identified hotspots in this area, which can guide future research directions. However, there are several limitations to consider. First, the data were extracted solely from the WOS database, and the search was restricted to English-language publications, focusing only on articles and reviews. Thus, some publications might be ignored in our analysis. Additionally, the publication type labels assigned by WOS may not be entirely accurate.

## CONCLUSION

Increasing evidence has demonstrated that astrocytes play an important role in depression and antidepressant treatment. However, systematic research in this field remains limited. In this study, we innovatively analyze the research resends of astrocytes in depression, based on the 2896 publications in this field from 1967 to 2024. The results of the bibliometric study prove neuroinflammation is a hot topic in this research field. Then, we review the role of astrocytes in neuroinflammation and summarize the evidence that astrocyte-mediated neuroinflammation is involved in the pathogenesis of depression and contributes to antidepressant treatment. In the future, more attention should be paid to the mechanisms underlying astrocyte-mediated neuroinflammation to develop potential antidepressant targets.

## Figures and Tables

**Fig. (1a):**
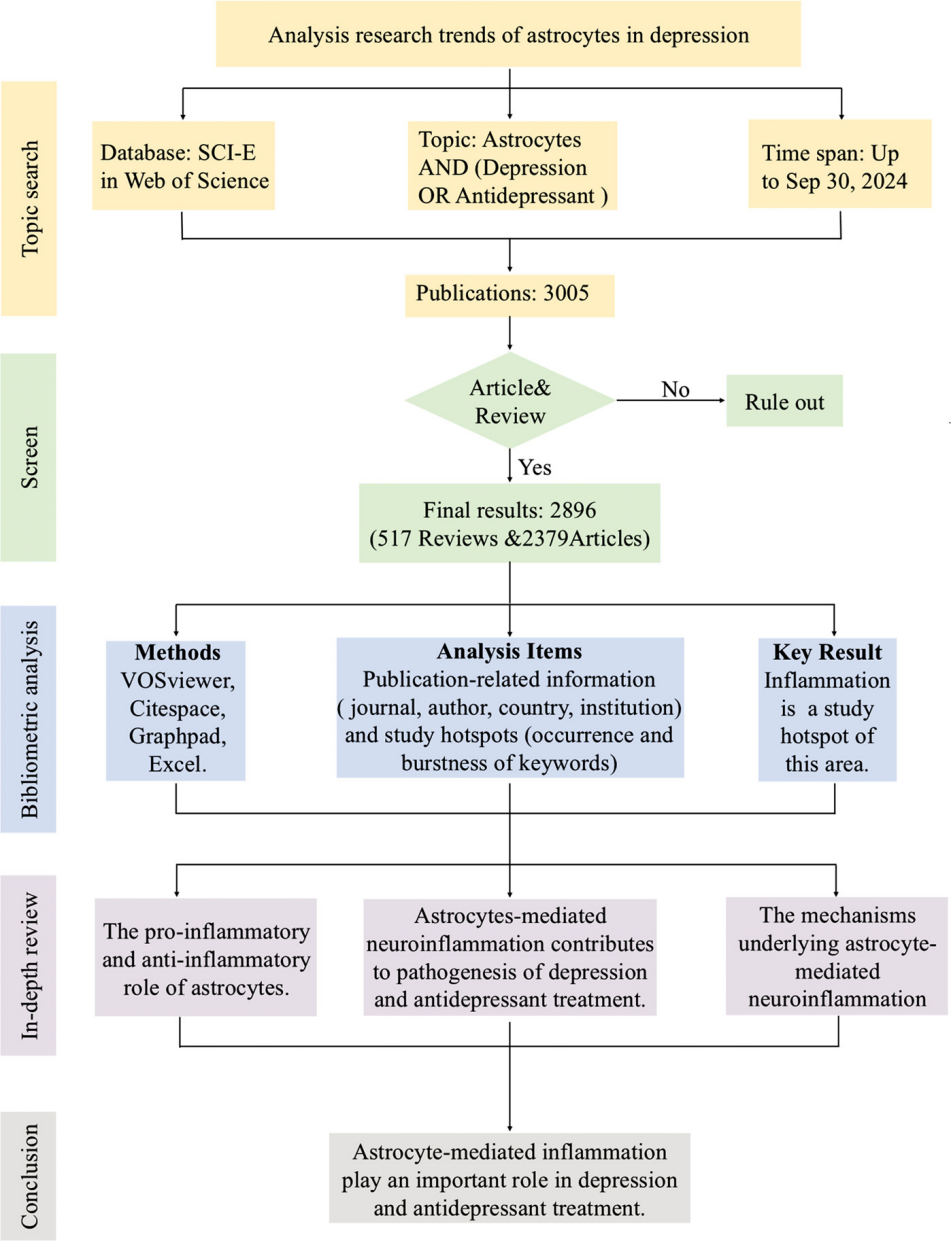
The flow chart of study of astrocytes in depression.

**Fig. (1b):**
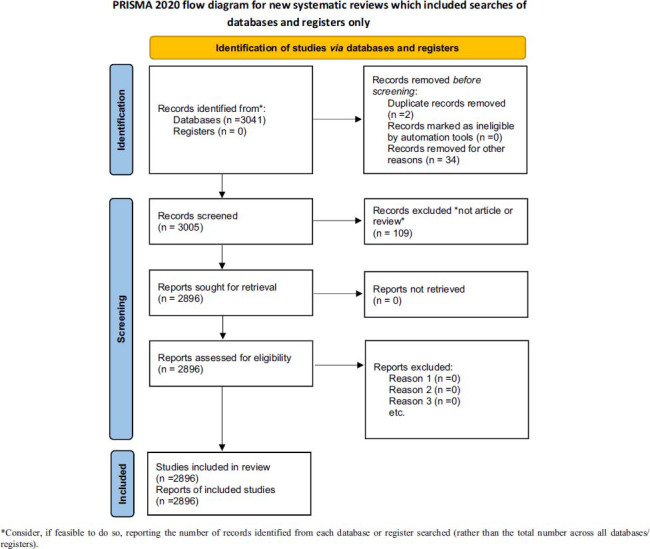
PRISMA flow diagram of the study.

**Fig. (2):**
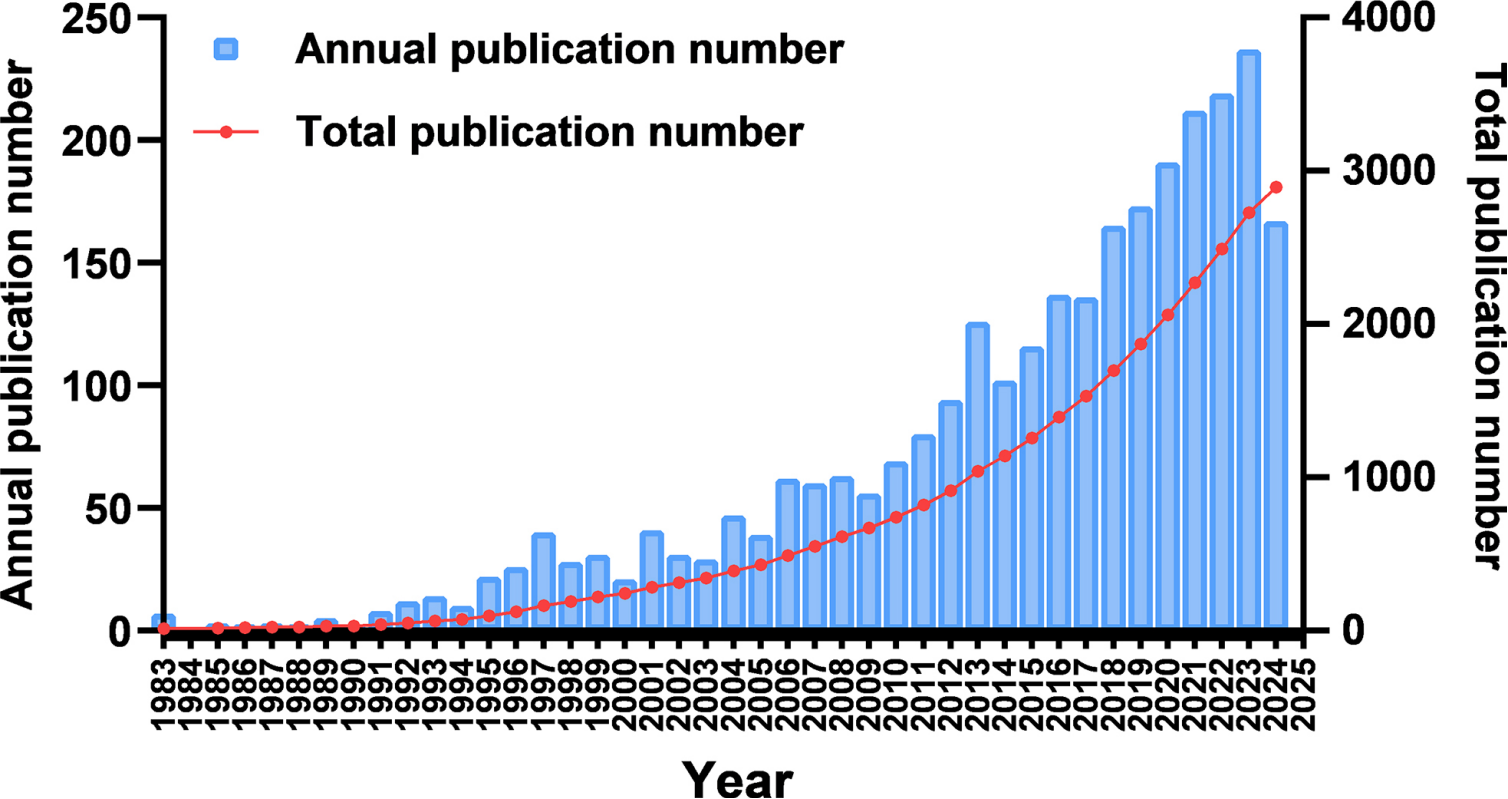
Publication trends of literature on astrocytes and depression up to September 30, 2024.

**Table 1:** Content of publication in the research field of astrocytes and depression.

**Content of Publication**		**Numbers of Publication**	**Percent (%)**
Clinic-relevant study and systematic review	Systematic Review	5	0.1727
	Systematic Review and Meta-analysis	7	0.2417
	Random Control Test	5	0.1727
	Cohort Study	3	0.1036
	Case-Control Study	104	3.5912
	Case-Series Study	22	0.7597
	Cross-Sectional Study	12	0.4144
	Case Report	6	0.2072
	Secondary-Analysis of Human Database	9	0.3108
Review (Exclude clinic-relevant systematic review)		509	17.58
Non-clinic study (*In vivo*, *in vitro*, *in silico* experiment, and others)		2214	76.45
Total		2896	100.00

**Table 2:** Publications with high citations in the research field of astrocytes and depression.

**Rank**	**Title**	**Journals**	**Year**	**Type**	**Citations**	**References**
1	Glial loss in the prefrontal cortex is sufficient to induce depressive-like behaviors.	*Biological Psychiatry*	2008	Article	527	[20]
2	Neuroinflammation and depression: Microglia activation, extracellular microvesicles, and microRNA dysregulation.	*Frontiers in Cellular Neuroscience*	2015	Review	466	[33]
3	Astrocyte-derived ATP modulates depressive-like behaviors.	*Nature Medicine*	2013	Article	455	[27]
4	Astrocytes pathology in major depressive disorder: Insight from human postmortem brain tissue.	*Current Drug Targets*	2013	Review	446	[16]
5	Pathogenesis of depression: Insights from human and rodent studies.	*Neuroscience*	2016	Review	418	[32]
6	Astroglial Kir4.1 in the lateral habenula drives neuronal bursts in depression.	*Nature*	2018	Article	403	[31]
7	Astroglial plasticity in the hippocampus is affected by chronic psychosocial stress and concomitant fluoxetine treatment.	*Neuropsychopharmacology*	2006	Article	401	[37]
8	Glial cell abnormalities in major psychiatric disorders: The evidence and implications.	*Brain Research Bulletin*	2001	Review	381	[38]
9	Markers of central inflammation in major depressive disorder: A systematic review and meta-analysis of studies examining cerebrospinal fluid, positron emission tomography and post-mortem brain tissue.	*Brain Behavior Immunity*	2019	Review	338	[35]
10	An inflammation-centric view of neurological disease: Beyond the neuron.	*Frontiers in Cellular Neuroscience*	2018	Review	322	[36]
11	Altered expression of glutamate signaling, growth factor, and glia genes in the locus coeruleus of patients with major depression.	*Molecular Psychiatry*	2011	Article	275	[39]
12	Glial fibrillary acidic protein immunoreactivity in the prefrontal cortex distinguishes younger from older adults with major depressive disorder.	*Biological Psychiatry*	2000	Article	262	[11]
13	Astrocytic abnormalities and global DNA methylation patterns in depression and suicide.	*Molecular Psychiatry*	2015	Article	236	[40]
14	Menin deficiency leads to depressive-like behaviors in mice by modulating astrocyte-mediated neuroinflammation.	*Neuron*	2018	Article	165	[41]
15	An astroglial basis of major depressive disorder? An overview.	*Glia*	2017	Review	161	[34]

**Table 3:** Top 10 productive journals in the research field of astrocytes and depression.

**Rank**	**Journal**	**Number of Publications**	**Number of Citations**	**Impact Factor**	**Journal Citation Indicator**
1	*Brain Research*	72	2455	2.7	0.7
2	*Glia*	66	3755	5.5	1.43
3	*International Journal of Molecular Sciences*	62	954	4.9	0.71
4	*Journal of Neuroscience*	62	9018	4.4	1.33
5	*Neuroscience*	59	3196	2.9	0.76
6	*Brain Behavior and Immunity*	56	3088	8.8	2.63
7	*Frontiers in Cellular Neuroscience*	56	2591	4.2	0.9
8	*Journal of Neurochemistry*	52	2344	4.2	0.83
9	*Neuropharmacology*	47	1725	4.6	1.19
10	*Journal of Neuroinflammation*	44	2677	9.3	1.93

**Table 4:** Core authors in the research field of astrocytes and depression.

**Authors**	**Organizations**	**Publications**	**Citations**	**H-index**
Li, BaoMan	China Medical University	31	1059	28
Verkhratsky, Alexei	University of Manchester	27	978	104
Peng, Liang	China Medical University	23	703	59
Chen, Nai-Hong	Chinese Academy of Medical Sciences - Peking Union Medical College	20	502	38
Araque, Alfonso	University of Minnesota	17	2946	29
Takebayashi, Minoru	Kumamoto University	17	471	22
Turecki, Gustavo	Kumamoto University	16	1191	22
Wang, Zhen-Zhen	Chinese Academy of Medical Sciences	16	299	18
Hisaoka-Nakashima, Kazue	Graduate School of Biomedical and Health Sciences, Hiroshima University	15	309	22
Hu, Gang	Nanjing University of Chinese Medicine	14	736	59

**Table 5:** Top 10 the most productive countries/regions in research field of astrocytes and depression.

**Rank**	**Country/Region**	**Publications**	**Citations**	**Total Link Strength**
1	USA	832	65575	486
2	China	677	18445	238
3	Germany	265	17512	239
4	Japan	251	10028	87
5	Canada	192	13241	180
6	England	159	10601	194
7	Spain	140	7173	159
8	Italy	115	7210	93
9	France	110	8143	116
10	Australia	94	4787	93

**Table 6:** Top 10 the most productive organizations in research field of astrocytes and depression.

**Rank**	**Organizations**	**Publications**	**Citations**	**Total Link Strength**
1	China Medical University	49	1405	71
2	Mcgill University	39	2302	40
3	University of Toronto	34	894	50
4	University of Manchester	33	1207	89
5	Consejo Superior de Investigaciones Cientificas	32	3733	27
6	Nanjing Medical University	32	1381	31
7	Huazhong University of Science Technology	30	1249	34
8	Karolinska Institute	29	1797	24
9	Southern Medical University	29	1071	31
10	Chinese Academy of Science	28	1261	63

**Table 7:** Characteristics of pro-inflammatory or anti-inflammatory astrocytes.

**Characteristics/Type**		**Pro-inflammatory Astrocytes**	**Anti-inflammatory Astrocytes**
Induced factor		LPS [50, 51]; IL-1β, TNF-α [53]; TNF-α/IL-1α [54]; IL-1α/TNF/C1q [52]; aging [63]	PK2 [64]; TGF-β1/BMP4 [56, 65]; FGFs [62, 66]; middle cerebral artery occlusion [57]
Morphology		Long dendrites [62]	Few dendrites [62]
Marker		C3 [52, 58-60]	S100A10 [67]
Secreted pro-inflammatory or anti-inflammatory molecules		TNF-α, IL-1β, IL-6, IL-8, IL-12, IL-23, GM-CSF, NO, iNOS [10, 50, 68], CXCL1, CXCL2, CCL2, CCL7, CXCL10 [10, 53, 54, 63]	IL-1ra, IL-10, IL-33, TGF-β, Arg1 [61, 69]
Interactions with other cells	Neuron	Impair neurons and induce neuronal apoptosis [70, 71].	Promote neural repair and increase neuronal complexity [72, 73].
	Microglia	Activate microglial and create a feed-forward loop of inflammation. [69, 74, 75].	Attenuate microglial activation through TGF-β/ IL-10 [76].
	Oligodendrocyte	Promote apoptosis of oligodendrocytes *via* TNF and Fas ligand [77].	Promote the transformation of oligodendrocyte precursor cells (OPCs) to mature oligodendrocytes through mitochondria migration [78].
The blood-brain barrier (BBB)		Impair BBB integrity [79].	BBB maintenance [80, 81].

## LIST OF ABBREVIATIONS

CNS: Central Nervous System
GFAP: Glial Fibrillary Acidic Protein
SCI-E: Science Citation Index Expanded
WHO: World Health Organization
WOS: Web of Science

## STANDARDS OF REPORTING

PRISMA guidelines and methodology were followed.

## SUPPLEMENTARY MATERIAL

PRISMA checklist is available as supplementary material on the publisher’s website along with the published article. Supplementary material is available on the publisher’s website along with the published article.
