# Supplementary material for: Research Trends and Evolution of Astrocytes in Depression and Antidepressant Treatment: A Bibliometric Analysis
Source: Curr Neuropharmacol. 2025 Mar 18;23(11):1405–22. doi: 10.2174/011570159X353752250227113751 (PMC12606658; doi:10.2174/011570159X353752250227113751)
Supplement: Supplementary file 1 [file CN-23-11-1405_SD1.pdf]

Supplementary Material

Research Trends and Evolution of Astrocytes in Depression and Antidepressant Treatment: A Bibliometric Analysis

Shu-Man Pan[1,#], Zhe Li[1,#], Jing-Qi Zhou[1], Xiang Shang[1], Tian-Jia Gu[1], Xiao-Ming Sun[1,*] and Zhen-Hua Zhu[1,*]`

[1]*Affiliated Guangji Hospital of Soochow University, Suzhou 215137, Jiangsu Province, P.R. China*

1875-6190/25 $65.00+.00 **© 2025 Bentham Science Publishers**

PRISMA 2009 Checklist

Section/topic	#	Checklist item	Reported on page #
**TITLE**			
Title	1	Identify the report as a systematic review, meta-analysis, or both.	Page 13, line 1 to 3
**ABSTRACT**			
Structured summary	2	Provide a structured summary including, as applicable: background; objecti-ves; data sources; study eligibility criteria, participants, and interventions; study appraisal and synthesis methods; results; limitations; conclusions and implications of key findings; systematic review registration number.	Page 14, line 30 to 49
**INTRODUCTION**			
Rationale	3	Describe the rationale for the review in the context of what is already known.	Page 14 and page 15, line 56 to 81
Objectives	4	Provide an explicit statement of questions being addressed with reference to participants, interventions, comparisons, outcomes, and study design (PI-COS).	Page 15, line 81 to 88
**METHODS**			
Protocol and registra-tion	5	Indicate if a review protocol exists, if and where it can be accessed (e.g., Web address), and, if available, provide registration information including registra-tion number.	Not mention
Eligibility criteria	6	Specify study characteristics (e.g., PICOS, length of follow-up) and report characteristics (e.g., years considered, language, publication status) used as criteria for eligibility, giving rationale.	Page 17, line 102 to 105.
Information sources	7	Describe all information sources (e.g., databases with dates of coverage, contact with study authors to identify additional studies) in the search and date last searched.	Page 16 and page 17, line 93 to 107.
Search	8	Present full electronic search strategy for at least one database, including any limits used, such that it could be repeated.	Page 16 and page 17, line 94 to 101.
Study selection	9	State the process for selecting studies (i.e., screening, eligibility, included in systematic review, and, if applicable, included in the meta-analysis).	Page 16, figure1. Page 17, line 110.
Data collection pro-cess	10	Describe method of data extraction from reports (e.g., piloted forms, indepen-dently, in duplicate) and any processes for obtaining and confirming data from investigators.	Page 17, line 109 to 117.
Data items	11	List and define all variables for which data were sought (e.g., PICOS, funding sources) and any assumptions and simplifications made.	Not applicable.
Risk of bias in indivi-dual studies	12	Describe methods used for assessing risk of bias of individual studies (inclu-ding specification of whether this was done at the study or outcome level), and how this information is to be used in any data synthesis.	Not applicable.
Summary measures	13	State the principal summary measures (e.g., risk ratio, difference in means).	Page 17, line 109 to 114.
Synthesis of results	14	Describe the methods of handling data and combining results of studies, if done, including measures of consistency (e.g., I^2) for each meta-analysis.	Not applicable. This work is not meta-analysis.

Section/topic	#	Checklist item	Reported on page #
Risk of bias across studies	15	Specify any assessment of risk of bias that may affect the cumulative evidence (e.g., publication bias, selective reporting within studies).	Not applicable.
Additional analyses	16	Describe methods of additional analyses (e.g., sensitivity or subgroup analyses, meta-regression), if done, indicating which were pre-specified.	Page 17, line 111 to 117.
**RESULTS**			
Study selection	17	Give numbers of studies screened, assessed for eligibility, and included in the review, with reasons for exclusions at each stage, ideally with a flow diagram.	Page 16, Figure 1. Besides, we have provided PRISMA 2020 flow diagram to editor office about three weeks ago.
Study characteristics	18	For each study, present characteristics for which data were extracted (e.g., study size, PICOS, follow-up period) and provide the citations.	Page 17 and page 18, line 119 to 134
Risk of bias within studies	19	Present data on risk of bias of each study and, if available, any outcome level assessment (see item 12).	Not applicable.
Results of individual studies	20	For all outcomes considered (benefits or harms), present, for each study: (a) simple summary data for each intervention group (b) effect estimates and confidence intervals, ideally with a forest plot.	Not applicable.
Synthesis of results	21	Present results of each meta-analysis done, including confidence intervals and measures of consistency.	Not applicable.
Risk of bias across studies	22	Present results of any assessment of risk of bias across studies (see Item 15).	Not applicable.
Additional analysis	23	Give results of additional analyses, if done (e.g., sensitivity or subgroup analyses, meta-regression [see Item 16]).	Page 19 to page 25, line 135 to 231
**DISCUSSION**			
Summary of evidence	24	Summarize the main findings including the strength of evidence for each main outcome; consider their relevance to key groups (e.g., healthcare providers, users, and policy makers).	Page 25, line 233 to 244
Limitations	25	Discuss limitations at study and outcome level (e.g., risk of bias), and at review-level (e.g., incomplete retrieval of identified research, reporting bias).	Page 32, line 412 to 415
Conclusions	26	Provide a general interpretation of the results in the context of other evidence, and implications for future research.	Page 32, line 416 to 425
**FUNDING**			
Funding	27	Describe sources of funding for the systematic review and other support (e.g., supply of data); role of funders for the systematic review.	Page 13, line 14 to 19

From: Moher D, Liberati A, Tetzlaff J, Altman DG, The PRISMA Group (2009). Preferred Reporting Items for Systematic Reviews and Meta-Analyses: The PRISMA Statement. PLoS Med 6(7): e1000097. doi:10.1371/journal.pmed1000097

For more information, visit: **www.prisma-statement.org**.

Table S1. Numbers of publication per year in the research field of astrocytes and depression.

Year	Publication per Year	Culmulative Publication	Year	Publication per Year	Culmulative Publication
1967	1	1	2002	31	316
1971	1	2	2003	29	345
1976	2	4	2004	47	392
1977	1	5	2005	39	431
1981	3	8	2006	62	493
1982	1	9	2007	60	553
1983	7	16	2008	63	616
1985	3	19	2009	56	672
1986	2	21	2010	69	741
1987	3	24	2011	80	821
1988	1	25	2012	94	915
1989	5	30	2013	126	1041
1990	2	32	2014	102	1143
1991	8	40	2015	116	1259
1992	12	52	2016	137	1396
1993	14	66	2017	136	1532
1994	10	76	2018	165	1697
1995	22	98	2019	173	1870
1996	26	124	2020	191	2061
1997	40	164	2021	212	2273
1998	28	192	2022	219	2492
1999	31	223	2023	237	2729
2000	21	244	2024	167	2896
2001	41	285			

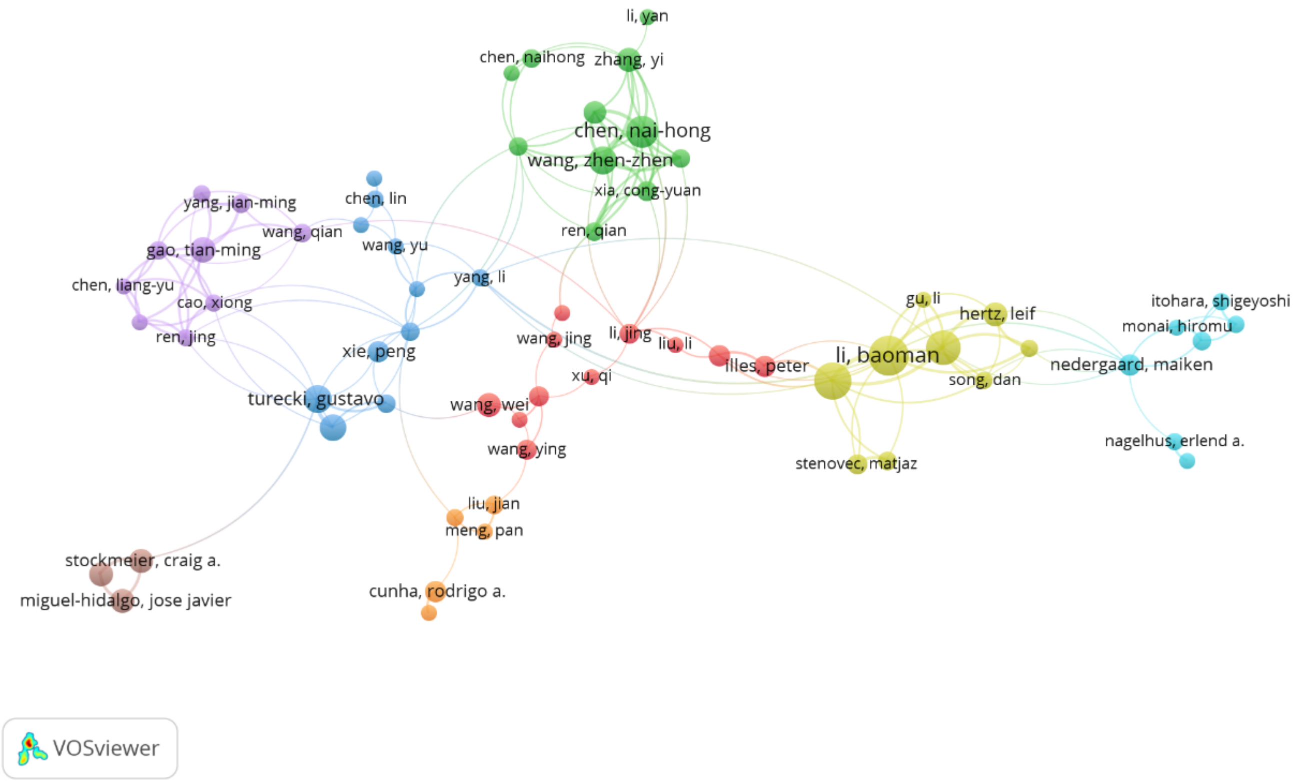

Fig. (S1). Visual analysis of authors' cooperation. Authors at least with 5 documents were analyzed. In this co-authorship map, the publication numbers of author are represented by node size.

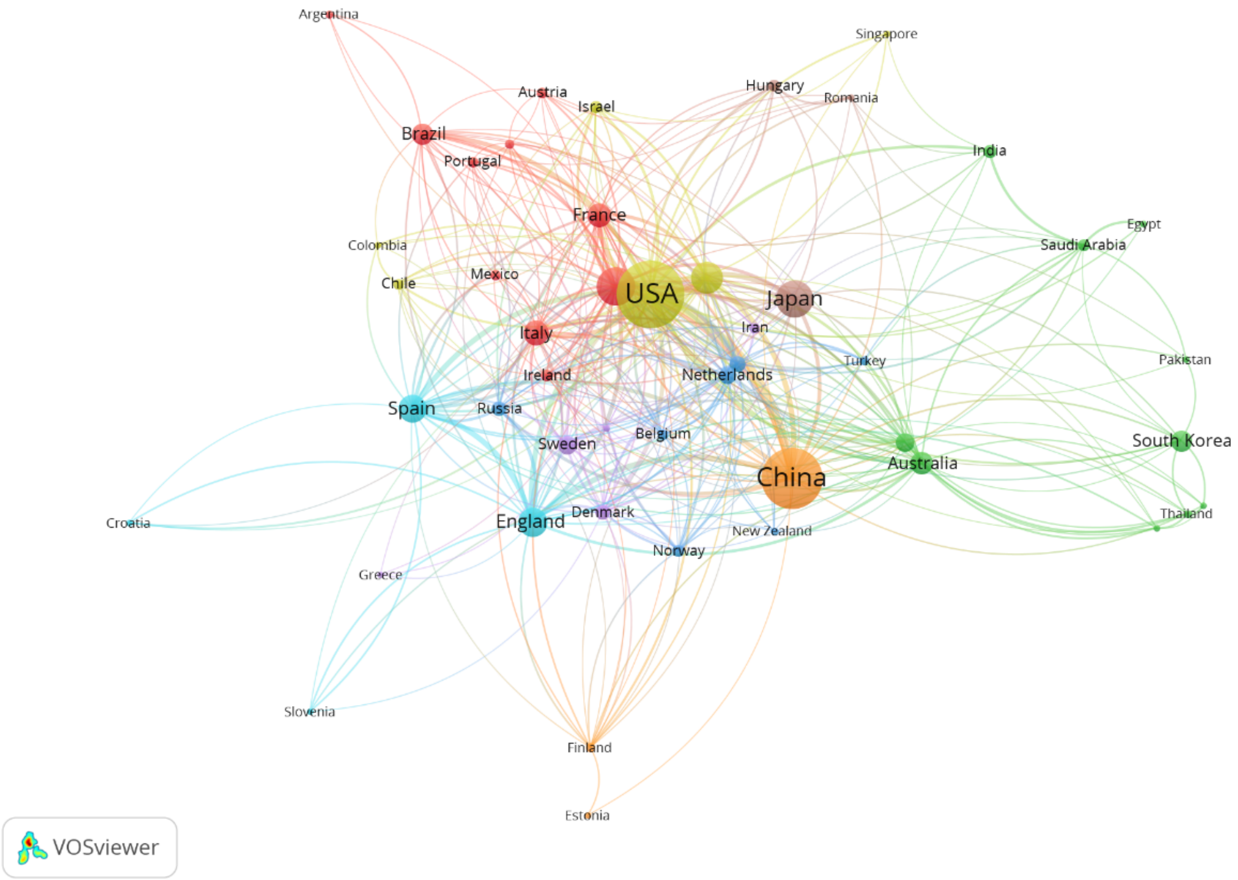

Fig. (S2). Visual analysis the cooperation of countries/regions. Documents co-authors more than 25 countries were ignored. Countries/regions with not less that 5 documents were selected. In our study, 47 countries/regions were selected after analysis.

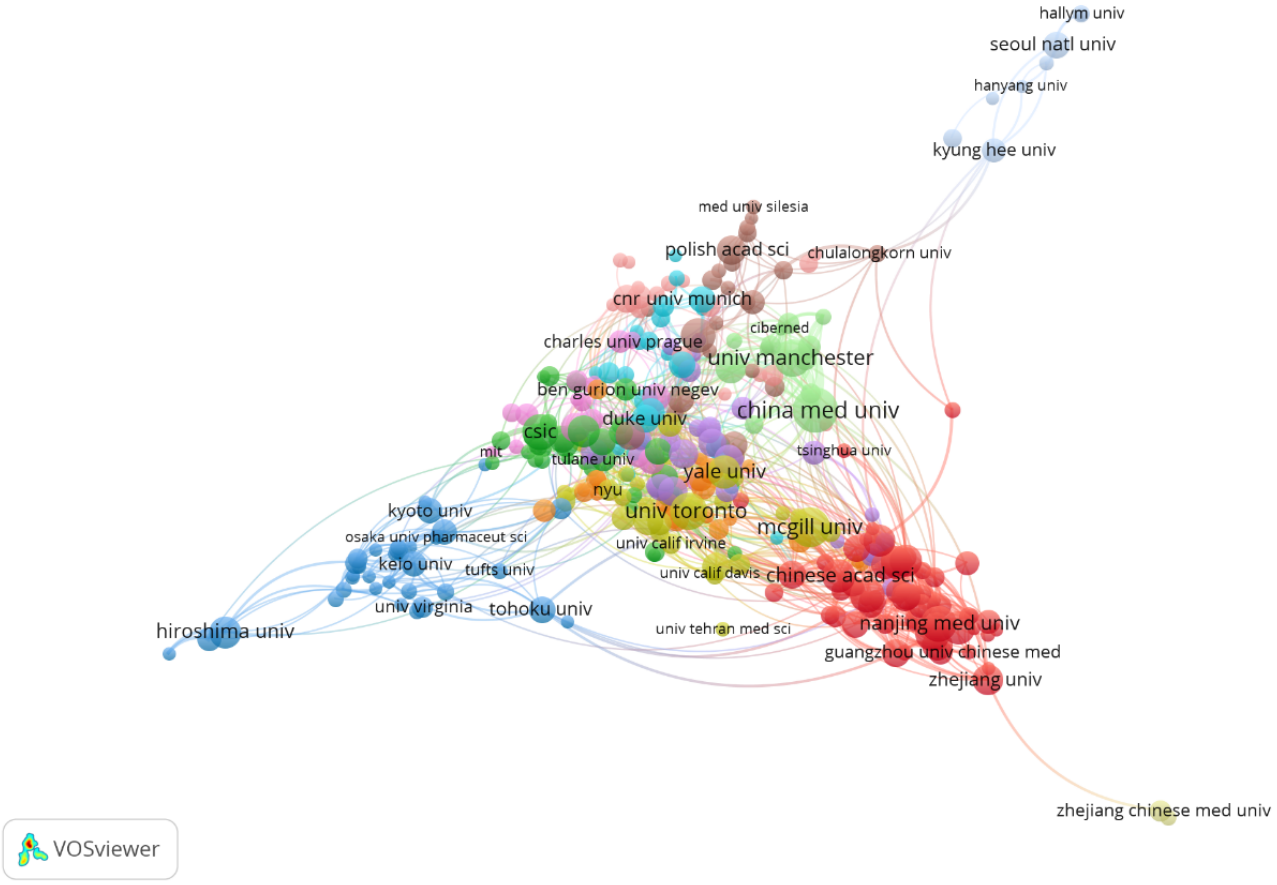

Fig. (S3). Visual analysis the cooperation of organizations. In this study, 340 organizations with not less than 5 documents were analyzed.
